# Supplementary material for: SUMOylation Potentiates ZIC Protein Activity to Influence Murine Neural Crest Cell Specification
Source: Int J Mol Sci. 2021 Sep 28;22(19):10437. doi: 10.3390/ijms221910437 (PMC8509024; doi:10.3390/ijms221910437)
Supplement: Supplementary file 1 [file ijms-22-10437-s001.zip › ijms-1387915-supplementary.pdf]

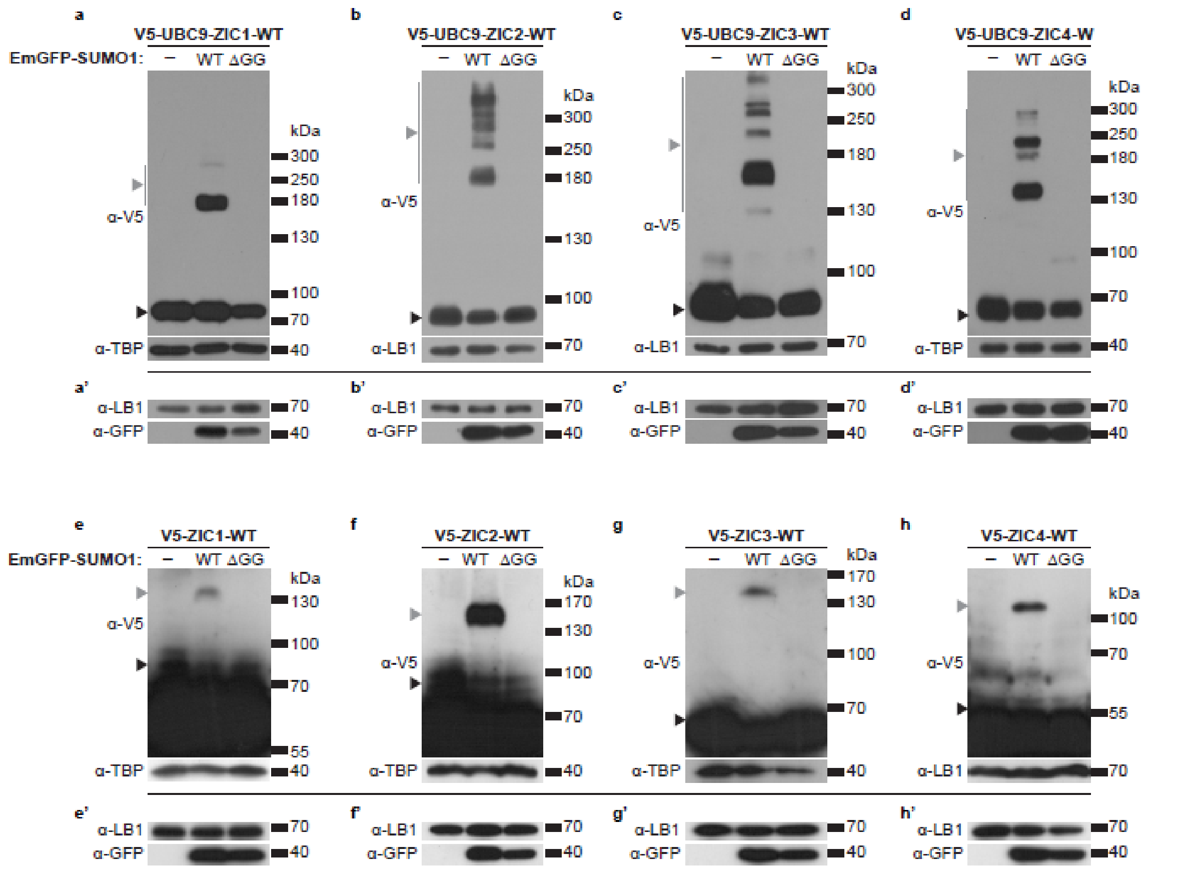

Figure S1. ZIC1-4 proteins are SUMOylated in HEK293T cells. **(a-d)** Representative WB of HEK293T cell nuclear fractions following transfection with the V5 epitope tagged, UBC9 fused ZIC expression plasmid shown, with or without EmGFP tagged SUMO1-WT or the conjugation defective EmGFP tagged SUMO1-ΔGG expression construct. A series of higher molecular mass, SUMO1-dependent forms are associated with each ZIC protein. N = 3 independent WBs. **(e-h)** Representative WB of HEK293T cell nuclear fractions following transfection with the V5 epitope tagged ZIC expression plasmid shown, with or without EmGFP tagged SUMO1-WT or the conjugation defective EmGFP tagged SUMO1-ΔGG expression construct. One higher molecular mass, SUMO1-dependent form is associated with each ZIC. N = 3 independent WBs. Grey arrow, SUMOylated ZIC; black arrow, basal ZIC. For WB of nuclear fractions (using antibodies against V5 or GFP) antibody against TBP or LAMIN B1 (LB1) was used as a loading control respectively, with the corresponding GFP WBs denoted a′ – h′.

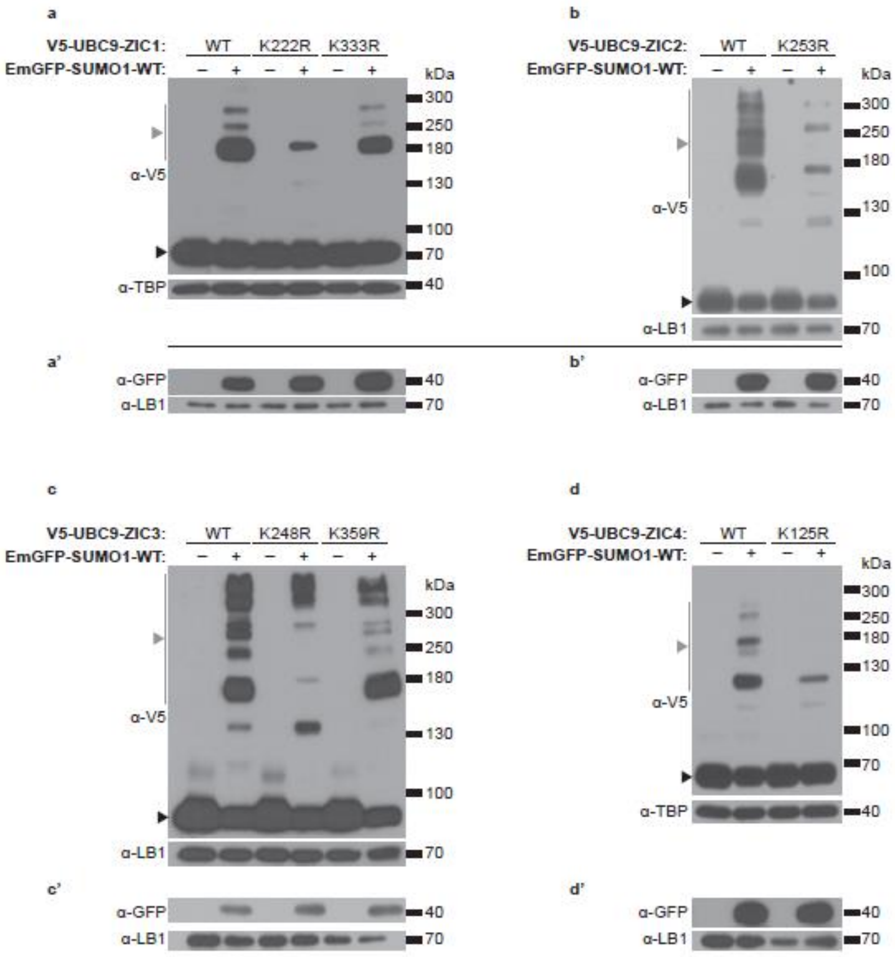

Figure S2. The UFDS system confirms the ZF-NC domain as the sole site of SUMO attachment for ZIC1-4 proteins. (a-d) Representative WB of HEK293T cell nuclear fractions following transfection with the V5 epitope tagged ZIC expression plasmid shown, with or without EmGFP tagged SUMO1-WT expression construct. (a) ZIC1, (b) ZIC2, (c) ZIC3 and (d) ZIC4. The SUMO-dependent higher molecular mass forms of each protein are substantially altered when the putative target lysine (K) within the ZF-NC (ZIC1 K222, ZIC2 K253, ZIC3 K248 and ZIC4 K125) is changed to an arginine (R), indicating these are a site of SUMO attachment in each protein. In contrast, the SUMO-dependent higher molecular mass forms of each protein are similar to those of the wild-type proteins when ZIC1 K333 or ZIC3 K359 are converted to the non-modifiable arginine residue, indicating that these lysines are not a SUMO-target. Grey arrow, SUMOylated ZIC; black arrow, basal ZIC. (a', b' c' d') WB to show overexpressed EmGFP-SUMO1-WT protein and corresponding loading control. For each protein, n = 3 independent transfections and WB. For WB of nuclear fractions (using antibodies against V5 or GFP), antibody against TBP or Lamin B1 (LB1) was used as a loading control respectively.

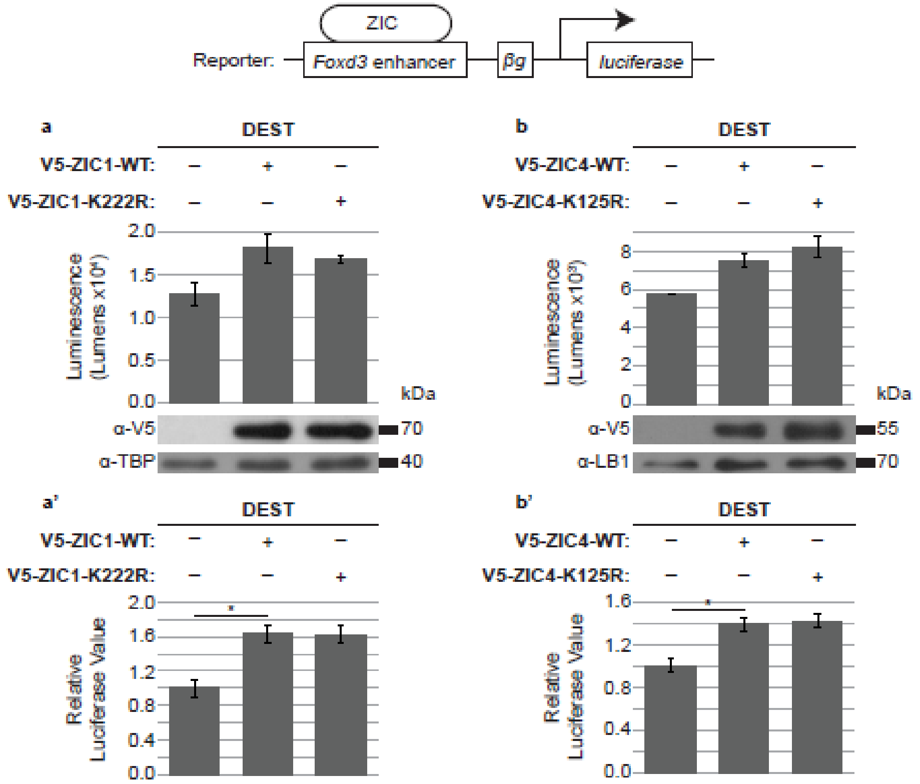

Figure S3. SUMOylation does not alter ZIC1 or ZIC4 activation of the Foxd3 enhancer. Luciferase activity at the Foxd3 reporter in HEK293T cells. (a and a′) The K222R SUMO-incompetent form of ZIC1 shows the same transactivation ability compared to wild-type ZIC1. (b and b′) The K125R SUMO-incompetent form of ZIC4 shows the same transactivation ability compared to wild-type ZIC4. (a, b) Raw data and WB showing overexpressed proteins from one representative experiment. For WB of nuclear fractions (using antibodies against V5) antibody against TBP or Lamin B1 (LB1) was used as a loading control. Error bars denote ±s.d. from three internal repeats. (a′, b′) Pooled data from three external repeats (normalised to background). Error bars denote ±s.e.m. (ANOVA). * : $p<0.05$, two way ANOVA with Bonferroni multiple comparison test.

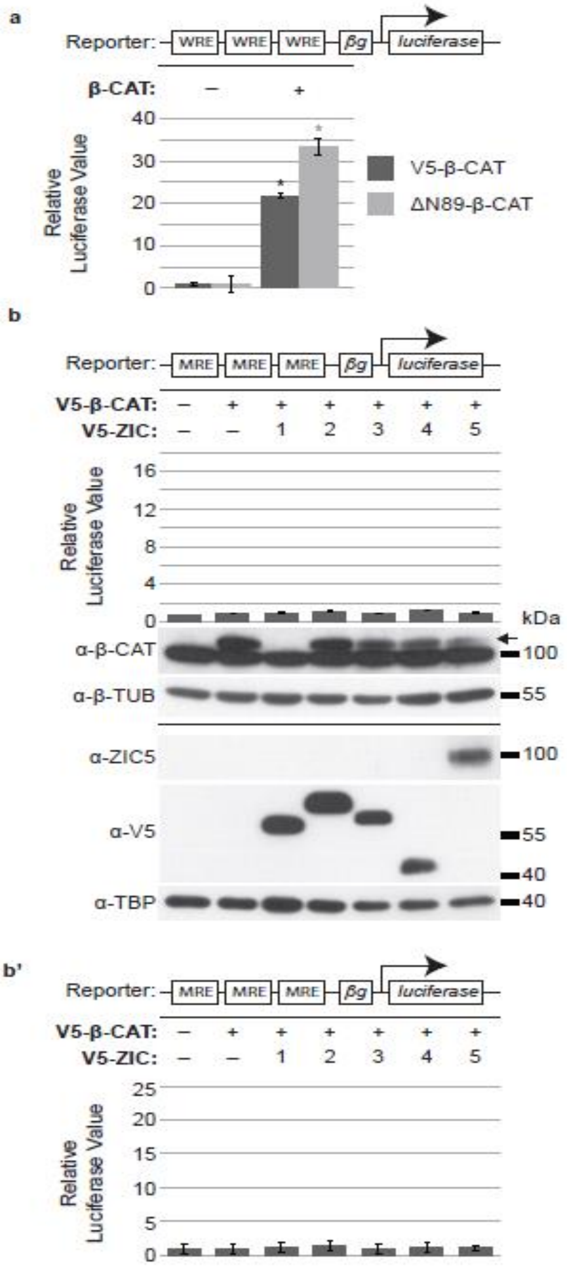

Figure S4. A ZIC optimised, TCF-dependent transcription assay. (a) Comparison of the reporter activity following transfection with wild-type b-catenin (V5-b-CAT) or stabilised b-catenin (ΔN89-b-CAT). Error bars denote ±s.e.m. of three external repeats, * : $p<0.05$, two-way ANOVA with Fischer's unprotected post ad hoc test. Black asterisk, V5-b-CAT data; grey asterisk, ΔN89-b-CAT data. Statistics to compare V5-b-CAT data and DN89-b-CAT data were not performed. (b and b') Neither bcatenin alone nor the co-expression of any ZIC proteins drives luciferase expression from the control reporter construct with mutated TCF binding motifs. (b) Raw data and WB showing overexpressed proteins from one representative experiment. For WB of cytoplasmic fractions (using antibody against b-catenin; a-b-CAT), antibody against b-tubulin (a-b-TUB) was used as a loading control. The arrow in the a-b-CAT WB denotes the larger, tagged exogenous protein. For WB of nuclear fractions (using antibody against V5 or sera against ZIC5) antibody against TBP was used as a loading control. Error bars denote ±s.d. from three internal repeats. Exogenous b-CAT is depleted to varying extents

in the presence of the different ZIC proteins, which is consistent with the observation that ZIC3 overexpression enhances b-catenin degradation [43,48]. (b′) Pooled data from three external repeats (normalised to background). Error bars denote ±s.e.m. (ANOVA).

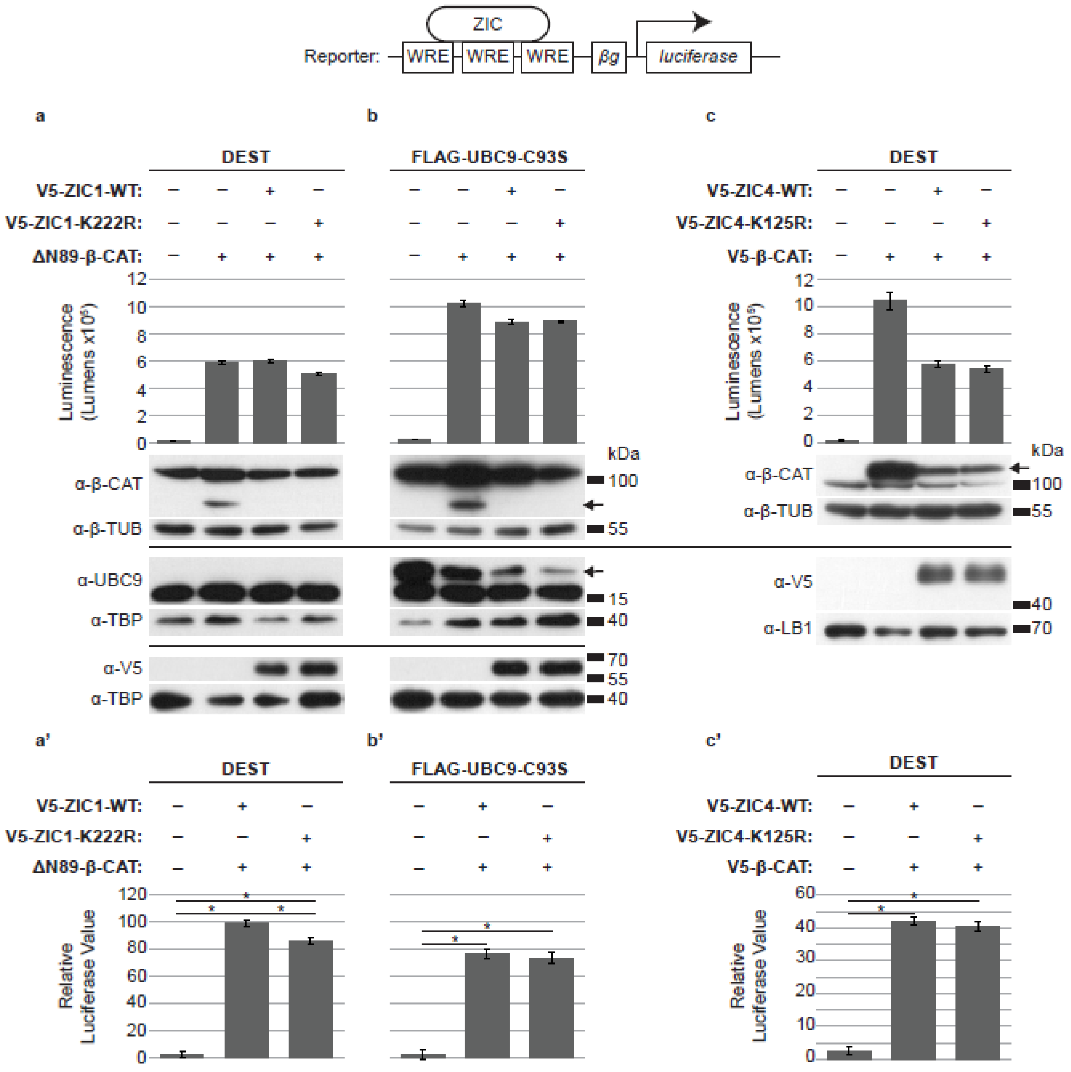

Figure S5. SUMOylation reduces ZIC1 but not ZIC4 inhibition of WNT signalling. Luciferase activity at the ZIC-optimised WRE reporter in HEK293T cells. (a and a′) The K222R SUMOincompetent form of ZIC1 shows a significant increase in inhibition of b-catenin-mediated

transcription compared to wild-type ZIC1. (b and b′) The ability of wild-type ZIC1 to inhibit b-cateninmediated transcription is increased when SUMOylation is universally inhibited via UBC9-C39S and is equivalent to the SUMO-incompetent form of ZIC1. (c and c′) The K125R SUMO-incompetent form of ZIC4 shows the same inhibition of b-catenin-mediated transcription as wild-type ZIC4. (a-c) Raw data and WB showing overexpressed proteins from one representative experiment. For WB of cytoplasmic fractions (using antibody against b-catenin; a-b-CAT), antibody against b-tubulin (a-b-TUB) was used as a loading control. The arrow in the a-b-CAT WB (a-c) denotes the tagged exogenous protein (V5-b-CAT is larger and ΔN89-b-CAT is smaller than the endogenous b-CAT). For WB of nuclear fractions (using antibody against V5 or UBC9) antibody against TBP was

used as a loading control. The arrow in the UBC9 WB denotes the larger, tagged exogenous protein. Error bars denote ±s.d. from three internal repeats. (a'-d') Pooled data from three external repeats (normalised to background). Error bars denote ±s.e.m. (ANOVA). * : $p<0.05$, two way ANOVA with Bonferroni multiple comparison test.
